# Severe cardiotoxicity in 2 patients with thymoma receiving immune checkpoint inhibitor therapy: A case report

**DOI:** 10.1097/MD.0000000000031873

**Published:** 2022-11-18

**Authors:** Shiwei Liu, Guikai Ma, Hui Wang, Guohua Yu, Jun Chen, Wenjing Song

**Affiliations:** a Joint Surgery Department, The First Affiliated Hospital of Weifang Medical University, Shandong, China; b Oncology Department, the First Affiliated Hospital of Weifang Medical University, Shandong, China; c Medical Oncology, The Second Hospital of Dalian Medical University, Liaoning, China.

**Keywords:** immune checkpoint inhibitors, immune myocarditis, immune-related adverse events, thymoma

## Abstract

Immune checkpoint inhibitors (ICIs) are currently approved for a variety of cancers and their use is expanding from advanced disease to first-line metastatic and adjuvant therapies. With the wide application of immunotherapy, its adverse reactions are also the object we need to pay attention to. Among its adverse events, immune myocarditis has low morbidity, but a high fatality rate. Simultaneously, the unique biological properties of thymic epithelial tumors (TETs) increase the risk of immune-mediated toxicity.

**Patient concerns:**

Patient 1 underwent chest computed tomography (CT) in April 2019 due to physical examination, which showed pleural metastasis of thymoma. Tissue puncture under CT guidance revealed type B2 thymoma. First-line chemotherapy with docetaxel combined with nedaplatin was administered, and apatinib was administered as a maintenance therapy after chemotherapy. After a regular review, progression of the disease was observed in April 12, 2021.

Patient 2 underwent anterior mediastinal tumor resection on August 2, 2019, due to the completion of the CT examination during myasthenia gravis to suggest a thymic tumor. Postoperative pathology revealed type B3 thymoma. The patient underwent local radiotherapy from October 2019 to November 2019. After irregular reexamination, the patient’s condition was stable. Disease progression has been observed in June 2021.

**Diagnosis:**

Both patients were diagnosed with thymoma.

**Interventions:**

Patient 1 was administered one cycle of gemcitabine, carboplatin, and sintilimab after disease progression. Patient 2 was treated with docetaxel and cisplatin for 2 cycles, and tislelizumab was added in the second cycle.

**Outcomes:**

Both patient 1 and patient 2 developed immune myocarditis after one cycle of immunotherapy. The difference was that patient 1 died within a few days. After a few days of active treatment for patient 2, the immune myocarditis did not improve significantly, and the patient chose to give up the treatment and go home. The shocking outcome is that the patient remains alive and stable.

**Lessons:**

Oncologists should be wary of ICI-related myocarditis owing to its early onset, nonspecific symptoms, and fulminant progression, especially when ICIs are used in combination. The patient’s cardiac condition should be assessed before administering ICIs.

## 1. Introduction

Thymic epithelial tumors (TETs) are a rare group of thoracic cancers, with an incidence of approximately 1.5 cases per million.^[1,2]^ According to the histopathological classification of the World Health Organization, TETs are classified as thymic carcinomas, thymomas, and thymic neuroendocrine carcinomas. Histologically, thymoma, unlike thymic carcinoma (epithelial carcinoma), tends to resemble a normal thymic structure and contains a mixture of epithelial tumor cells and non-tumor lymphocytes. Thymoma is frequently associated with paraneoplastic autoimmune disease (AD) due to an underlying defect in immune tolerance.^[3]^ The most common form of AD is myasthenia gravis, which occurs in 30% to 50% of patients with thymoma.^[4]^

Immune checkpoint inhibitors (ICIs) targeting programmed death 1/ligand 1 (PD-1/PD-L1) and cytotoxic T-lymphocyte antigen 4 (CTLA-4) have transformed the therapeutic landscape of many cancers and have shown significant efficacy in multiple tumor types.^[5,6]^ The toxic effects related to ICIs may affect any organ and arise from the activation of autoreactive T cells that destroy host tissues. Most commonly, these immune-related adverse events (IRAEs) affect the lung, liver, colon, thyroid, pituitary, and skin, but rare events involving the nervous system, heart, and other organs also occur.^[7,8]^ Thymus is a lymphatic organ in the development of the immune system and may contribute to the high incidence of IRAEs induced by ICI therapy.^[9]^ In this rare thoracic tumor, the use of ICIs should be carefully considered and closely monitored. The reported incidence of ICI-related myocarditis is 0.04% to 1.14%, compared with other IRAEs, it has a significantly higher mortality rate of 25% to 50%.^[10–12]^ In addition, the incidence and mortality of myocarditis with combined ICIs were nearly double those of other IRAEs, although it remains a rare adverse event.^[13,14]^ Currently, there are limited data on the risk factors, specific clinical manifestations, laboratory tests and examination characteristics, and treatment and outcomes of immune myocarditis. Therefore, it is necessary to better describe immune-related myocarditis. Here, we introduce the detailed data of 2 cases of thymoma with immune myocarditis during the application of ICIs and summarize some insights from them.

## 2. Case reports

### 2.1. Case 1

**Figure 1.**
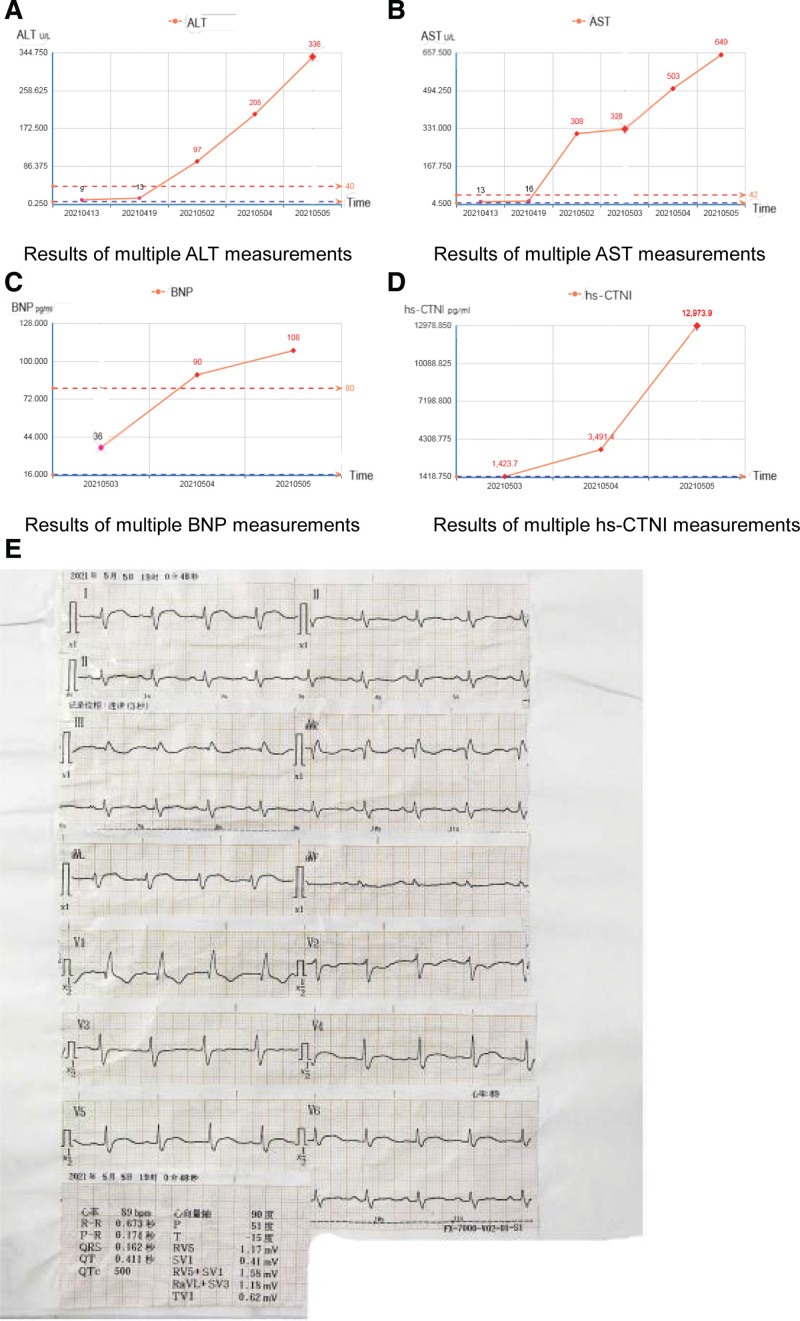
The changes of ALT, AST, BNP, hs-CTNI and the electrocardiogram of patient 1 during treatment. (A) Results of multiple ALT measurements, (B) results of multiple AST measurements, (C) results of multiple BNP measurements, (D) results of multiple hs-CTNI measurements; and (E) this picture shows an ECG taken on May 5, 2021. At this time, ECG had already suggested incomplete right bundle branch block, ST-T changes, and QTc interval prolongation. ALT = alanine aminotransferase, AST = aspartate aminotransferase, BNP = brain natriuretic peptide, hs-CTNI = high-sensitivity troponin, ECG = electrocardiogram.

Patient 1, male, 42 years old, underwent chest computed tomography (CT) for physical examination in April 2019, which suggested pleural metastasis of the thymoma. Tissue puncture under CT guidance suggested type B2 thymoma. From April 2019 to July 2019, four cycles of docetaxel and nedaplatin chemotherapy were administered, and the efficacy evaluation was stable. Following chemotherapy, apatinib (250 mg/day) was administered. The patient was given irregular oral medication and reexamination, and the condition was stable. On April 12, 2021, he was admitted to our department because of “persistent pain in the left shoulder for one month,” and underwent a CT scan of the chest, abdomen, and pelvis: a malignant mass in the anterior mediastinum with mediastinal lymph nodes, left pleura, left diaphragm, and bilateral lung metastasis, slightly larger lymph node on the left side of the heart, and involvement of the left pericardial wall. Brain magnetic resonance imaging (MRI) and bone scintigraphy revealed no signs of metastasis. On April 15, 2021, pleural puncture was performed. Postoperative pathology revealed type B3 thymoma. Immunohistochemical results: phosphocreatine kinase (CK) broad (+), CK5/6 (+), P63 (+), CK7 (partial +), CK19 (weak +), CD20 (lymphocytes +), WT-1 (-), Calretinin (-), CD5 (sporadic +), CD117 (-), TdT (sporadic +), PD-1 (-), PD-L1 (tumor cells +, 99%; immune cells +, 1%), MSH6 (+), PMS2 (+), MLH1 (+), MSH2 (partial +), CD56 (-), Syn (-), CgA (-), Ki-67 (15%). On April 17, 2021, one cycle of gemcitabine, carboplatin, and sintilimab chemotherapy was administered. On May 2, 2021, he was admitted to our department due to “fever for three days.” The patient developed fever on April 30, 2021, with a maximum temperature of 39.0 °C, accompanied by chest tightness and suffocation, no chest pain, rapid heart rate (110 beats/min), no cough expectoration, no abdominal pain, and diarrhea. Complete relevant tests and inspections, D dimer (D-D):8.58 ug/mL, macrobiochemical and myocardial enzyme spectrum: alanine aminotransferase (ALT) 97 U/L, aspartate aminotransferase (AST) 308 U/L, lactate dehydrogenase (LDH) 805 U/L, α-hydroxybutyrate dehydrogenase (HBDH) 549 U/L, CK 7603 U/L, creatine kinase isoenzyme mass (CK-MB) 53.37 ng/mL, high-sensitivity troponin (hs-CTNI) 1423.7 pg/mL. Interleukin (IL-6) 102.60 pg/mL, C-reactive protein (CRP) 71.1 mg/L, and procalcitonin (PCT) 0.530 ug/L. Routine blood tests revealed that the white blood cells and neutrophils were normal, the percentage of neutrophils was 88.9%, the percentage of neutrophils increased under a microscope, the cytoplasmic granules were coarsely stained, and the brain natriuretic peptide (BNP) level was normal. Lymphocyte subset analysis: CD3+CD4+/CD3+CD8 + 3.67, CD3+CD4 + 61.6%, CD3-CD(16 + 56) + 4.5%, CD3+CD8+ absolute value 218/uL, CD3-CD(16 + 56) + absolute value 59/uL. Electrocardiogram (ECG) showed sinus tachycardia (121 beats/min) and low voltage of the QRS complex in the limb leads. May 3, 2021 Echocardiography: EF67%; pericardial effusion (small amount). The test results after admission showed that liver function transaminase, CK, LDH, CK-MB, and hs-CTNI levels were significantly increased. At present, the BNP level was normal. After questioning the patient’s medical history, the patient had a history of heavy physical labor (carrying goods), and the weather had changed rapidly in recent days. The patient’s immunity was low after chemotherapy, and fever caused by the patient’s infection was not excluded, which further led to immune myocarditis. Consultation of the cardiology, ICU, and rheumatology departments was urgently needed to assist in diagnosis and treatment, and the possibility of immune myocarditis was considered. Methylprednisolone 1000 mg (May 3, 2021 to May 5, 2021), human immunoglobulin 30 g (May 5, 2021) pulse therapy, coenzyme Q10 nutrition myocardial therapy, metoprolol tablets (May 4, 2021 to May 5, 2021) heart rate lowering therapy, spironolactone tablets, furosemide diuretic therapy, reduced glutathione, magnesium isoglycyrrhizinate, bicyclol tablets liver protection therapy, and piperacillin tazobactam anti-infective therapy. On May 5, 2021, the patient’s general condition was poor, complaining of difficulty in breathing and eating, and the bulbar conjunctiva was edematous. Because the patient had a thymoma, muscle weakness was not excluded, and the oxygen saturation was 98% during nasal cannula inhalation. Blood gas analysis indicated type II respiratory failure, and a noninvasive ventilator was used to assist breathing. The reexamination of liver function transaminases still continued to increase, ALT 336 U/L, AST 649 U/L, immune hepatitis was not excluded, he had been given liver protection and methylprednisolone pulse therapy; the patient’s myocardial enzymes and hs-CTNI continued to increase, LDH 1718 U/L, HBDH 1259 U/L, CK 10802 U/L, CK-MB 272.6 ng/mL, hs-CTNI 12973.9 pg/mL, greatly increased, BNP 108.00 pg/mL, and May 5, 2022 ECG: sinus rhythm (89 beats/min), incomplete right bundle branch block, ST-T changes, QTc interval prolongation. May 5, 2021 Echocardiography: EF60%. Today’s ECG has changed compared with previous days, and cardiac bioelectrophysiology has been affected. It is recommended that the patient be transferred to the ICU or Department of Respiratory Intensive Medicine for continued treatment. The patient’s family refused to transfer the patient for economic reasons. On May 5, 2021, the patient suddenly became unconscious at 23:44, did not respond to calls, could not measure blood pressure, and had a weakened heartbeat and breathing. Continuous chest compressions were immediately administered, and symptomatic treatment such as epinephrine was administered to stimulate the heart and nicotine to stimulate breathing. Emergency consultation with the anesthesiology and intensive medicine departments revealed that the patient’s blood oxygen saturation continued to drop, and tracheal intubation ventilator-assisted ventilation was provided after consultation in the anesthesiology and intensive medicine departments. The patient’s heart rate progressively decreased, and blood pressure remained undetectable. The patient was administered epinephrine and other cardiotonic symptomatic treatments again, but the vital signs did not improve. The patient’s family indicated that they gave up continuing rescue and signed a notice to give up medical treatment. The patient died at 0:54 on May 6, 2021. In Figure 1, we show the ALT, AST, BNP, hs-CTNI, and ECG of patient 1 during treatment.

### 2.2. Case 2

**Figure 2.**
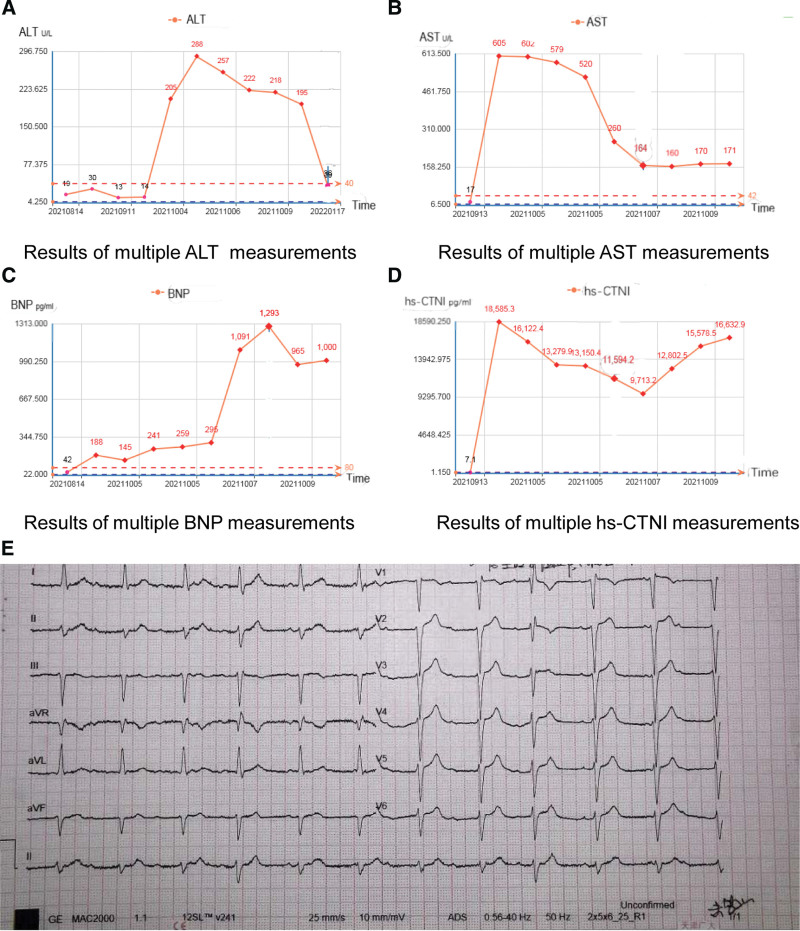
The changes of ALT, AST, BNP, hs-CTNI and the electrocardiogram of patient 2 during treatment. (A) Results of multiple ALT measurements, (B) results of multiple AST measurements, (C) results of multiple BNP measurements, (D) results of multiple hs-CTNI measurements; and (E) this picture is an ECG on October 7, 2021. At this time, ECG showed an accelerated junctional escape rhythm, atrioventricular block (occasionally sinus), intraventricular block, QTC interval > 470 ms, and increasing r-wave in the chest leads bad. ALT = alanine aminotransferase, AST = aspartate aminotransferase, BNP = brain natriuretic peptide, hs-CTNI = high-sensitivity troponin, ECG = electrocardiogram.

Patient 2, male, 52 years old, in August 2019, had slurred speech, difficulty swallowing, and general weakness without obvious reasons or incentives. On August 3, 2019, he was admitted to the Affiliated Hospital of Weifang Medical College and was diagnosed with myasthenia gravis. The effect of treatment was considered acceptable. During this period, CT was performed to identify a thymic tumor. The patient was admitted to our hospital on August 13, 2019. After completing relevant examinations, the patient underwent anterior mediastinal tumor resection. During surgery, there was a hard mass in the left anterior mediastinum, approximately 5 × 3 × 3 cm, with an incomplete capsule and a relatively fixed base. In addition, there was obvious external invasion on the surface of the left lung pleura, and the surface of the visceral pleura was scattered with small nodules. A piece of the pleural nodule was taken during the operation. Rapid pathological examination revealed malignant tumors, and the possibility of an invasive thymoma and malignant mesothelioma was high. Intraoperative diagnosis of thymic carcinoma with extensive pleural metastasis. Partial resection of the primary tumor in the mediastinum was performed and the pleural nodules were coagulated, cauterized, and washed with distilled water and lobaplatin (50 mg). Postoperative pathology revealed a thymoma (type B3, volume 5 × 3 × 0.5 cm). The postoperative recovery was good, and myasthenia gravis was significantly relieved. Subsequently, the patient underwent local radiotherapy from October 2019 to November 2019. After irregular reexamination, the patient’s condition was stable. In June 2021, the patient experienced persistent pain and discomfort in the left quarter-rib area. On August 5, 2021, an outpatient CT scan of the chest, abdomen, and pelvis showed that after thymic carcinoma, the left anterior mediastinal nodule and left pleura showed nodular thickening and metastasis. After admission to our department, CT-guided pleural puncture biopsy was performed on August 16, 2021. The postoperative pathological report showed: (left pleural puncture tissue) combined with a medical history consistent with thymoma, type B3. Immunohistochemical results: CK broad (+), Vimentin (partial +), P63 (+), CK5/6 (+), TdT (partial +), CD5 (partial +), CD117 (A little +), CK7 (individual +), CD3 (partial +), CD20 (a small amount +), CD1a (partial +), CD99 (partial +), Calretinin (a little +), MLH1 (+), MSH2 (+), MSH6 (+), PMS2 (+), Ki-67 index (60%). PD-L1(22C3)CPS = number of PD-L1 positive cells (tumor cells, lymphocytes, and macrophages)/total number of viable tumor cells × 100 = 55. Subsequently, according to the patient’s condition, diagnosis, and treatment guidelines, after discussion in the department, it was recommended that the patient combine immunotherapy with chemotherapy; however, this patient refused. From August 18, 2021, the patient was treated with one cycle of docetaxel and cisplatin, and the process proceeded smoothly. In the second cycle, the patients and their families requested additional immunotherapy; therefore, from September 11, 2021, they were treated with docetaxel d1, cisplatin d2, and tislelizumab 200 mg d3 in one cycle. Myocardial enzyme spectrum, hs-CTNI, BNP, and liver function were all normal before drug administration. On September 13, 2021, the laboratory test BNP 188.00 pg/mL, myocardial enzyme spectrum: LDH 245 U/L, HBDH 192 U/L, hs-CTNI and liver function were normal. On October 1, 2021, the patient underwent cervicothoracic, abdominal, and pelvic CT and cranial MRI to evaluate the condition, and the efficacy was evaluated by partial response (PR). The lung lesions and left pleura gradually reduced. The patient complained of chest tightness and suffocation after reexamination, and he did not experience any other discomfort. The laboratory examination results after admission were October 4, 2021. ALT 205 U/L, AST 605 U/L, LDH 1388 U/L, HBDH 1339 U/L, CK 9290 U/L, CK-MB 146.87 ng/mL, hs-CTNI 18585.3 pg/mL, BNP 145.00 pg/mL, the patient was given an ECG showing complete right branch block. Urgently seek consultation from the Department of Cardiology and Critical Care Medicine, consider immune myocarditis and immune hepatitis, give liver-protective drugs such as glutathione and magnesium isoglycyrrhizinate; give vitamin C, coenzyme Q10, and creatine phosphate sodium to nourish the myocardium; and recombinant human brain natriuresis peptide and isoproterenol pump therapy. According to the 2020 version of the Chinese expert consensus on the monitoring and management of immune checkpoint inhibitor-related myocarditis, the patient was given methylprednisolone 1 g qd pulse therapy for 3 to 5 days (October 5, 2021 to October 10, 2021), and human immunoglobulin were given at the same time (20 g qd*3d and 10 g qd*2d, October 5, 2021 to October 10, 2021). To prevent gastrointestinal bleeding, secondary fungi, bacteria, pneumocystis pneumonia, osteoporosis, deep vein thrombosis caused by high-dose hormone application, compound trimoxazole, cimetidine, and potassium citrate were added. October 6, 2021 Myocardial enzyme spectrum: LDH 1458 U/L, HBDH 1400 U/L, CK 3365 U/L, CK-MB 136.46 ng/mL, hs-CTNI 11594.2 pg/mL, BNP 295.00 pg/mL. October 7, 2021 Myocardial enzyme spectrum: AST 164 U/L, LDH 1212 U/L, HBDH 1303 U/L, CK 1842 U/L, CK-MB 105.07 ng/mL, hs-CTNI 9713.2 pg/mL, BNP 1091.00 pg/mL, 2021-10-07 ECG: accelerated junctional escape rhythm, atrioventricular block (occasionally sinus), intraventricular block, QTC interval > 470 ms, and increasing r-wave in the chest leads bad. The patient’s myocardial enzyme spectrum was lower than before, and the BNP level was significantly increased. The patient was considered to have acute heart failure, and torsemide 20 mg bid was added to diuretic treatment. October 8, 2021 Liver function: ALT 222 U/L, AST 160 U/L, BNP 1293.00 pg/mL, myocardial enzyme spectrum: LDH 1109 U/L, HBDH 1214 U/L, CK 1879 U/L, CK-MB 110.26 ng/mL, hs-CTNI 12802.5 pg/mL. The patient’s myocardial enzyme spectrum continued to rise over the past 2 days, and the patient’s symptoms did not improve significantly. Considering that the current treatment effect is is poor, mycophenolate mofetil 1.0 g bid was added according to the guidelines. October 10, 2021 Liver function: ALT 195 U/L, AST 171 U/L, LDH 1070 U/L, HBDH 1075 U/L, CK 1244 U/L, CK-MB 115.92 ng/mL, hs-CTNI 16632.9 pg/mL. After symptomatic treatment, the patient’s myocardial enzyme spectrum decreased in a short time, and liver function improved slightly. From October 8, 2021, the patient’s myocardial enzyme spectrum and BNP increased sharply again, and he was administered mycophenolate mofetil 1 g bid orally several times during the period. Dynamic monitoring of the ECG changes indicated that the atrioventricular block had worsened. After active continuous ECG monitoring, oxygen inhalation, myocardial nutrition, cardiotonics, diuretics, liver protection, and other drug treatments, the patient remained palpitated, chest tight, and suffocated. Progressive aggravation, inability to lie down, right eyelid drooping. On October 10, 2021, the patient’s family requested automatic discharge after careful consideration. After discharge, the patient received oral methylprednisolone 150 mg/day, a weekly dose reduction of 20 mg, mycophenolate mofetil 1 g bid, coenzyme Q10 nutrition for cardiac muscle, spironolactone diuretic treatment, glycyrrhizic acid diamine enteric-coated capsules, and bicyclol tablets for liver protection. The patient stopped mycophenolate mofetil on November 1, 2021, and the hormone was gradually reduced. On January 17, 2022, the patient’s liver function returned to normal. Since the patient did not reexamine troponin level, his specific condition was unknown, and reexamination CT showed that the tumor was in a stable condition. However, to date, the patient remains alive and in good physical condition, with an ECOG score of 2. In Figure 2, we show the ALT, AST, BNP, hs-CTNI, and ECG of patient 2 during treatment.

## 3. Discussion

From these 2 patients, we found the following common characteristics: both patients had thymoma, further verifying that patients with thymoma were more likely to develop IRAEs. Both patients were middle-aged male patients, relatively younger, and the younger the patient, the faster the disease progression, which is inconsistent with a previous report that elderly patients are more prone to ICIs-related myocarditis.^[13]^ Here we believe that the thymus structure and function of young patients are more normal, and it is easier to induce IRAEs. However, this needs to be confirmed by further research. At the same time, patient 2 underwent partial resection of the primary tumor in the mediastinum, and it is unknown whether the disease progression of patient 2 was slower than that of patient 1. All patients developed immune myocarditis after one cycle of ICI, with early onset and different clinical manifestations. Patient 1 mainly complained of fever, and patient 2 mainly complained of chest tightness and suffocation. Common features of both were chest tightness and suffocation. The expression of PD-L1 in the initial pathological diagnosis of these 2 patients was high. Patient 2 had a better curative effect after ICI treatment, and the curative effect was evaluated as PR. When hs-CTNI is significantly increased, it is often accompanied by an increase in liver transaminase levels. Therefore, attention should be paid to changes in liver function when monitoring hs-CTNI in early stages. Patients often have ECG changes in the early stages, which occur earlier than echocardiography, and the changes in cardiac ejection fraction have hysteresis. Therefore, ECG changes should be closely observed in patients who use ICIs. However, there is no unified standard for monitoring the intervals of ECG, hs-CTNI, and myocardial enzymes, and further research is required. Once a patient develops grade 3/4 immune myocarditis, it is difficult to reverse the patient’s condition even if high-dose glucocorticoids and immunosuppressants such as mycophenolate mofetil are administered in a timely manner, which is a huge challenge. However, there are individual differences. For the 2 cases in this study, the outcome of patient 2 was unexpected, and the durability of the effect of ICI was also fully reflected in patient 2. The treatment of ICIs and myocarditis is promising.

The role of immunotherapy in the treatment of a variety of cancers is rapidly expanding, owing to its durable clinical activity and good tolerability. Patients with TET are at increased risk of immune-mediated toxicity and are more prone to musculoskeletal and neuromuscular adverse events following immunotherapy. The development of AD in patients with TET, particularly thymoma, poses a major challenge for the development of ICIs. AD patients are often excluded from clinical trials evaluating ICI due to concerns of severe immunotoxicity.^[15]^ This exclusion is particularly strict for patients with thymoma, as the disease is often related to paraneoplastic autoimmunity.^[2]^ Fatal toxic effects related to ICIs are uncommon and have advantages over other oncological interventions, but occur in 0.3% to 1.3% of patients. These events typically occur early after treatment initiation and should not prevent the use of potentially curative therapies. Our understanding of ICI myocarditis is limited, and further analysis will help determine whether broad awareness prevents these lethal events.

Myocarditis should be diagnosed in the absence of other primary diagnoses of cardiac disease (e.g., chronic ischemic heart disease with or without heart failure and acute coronary syndrome). Elevated serum hs-CTNI and BNP levels are the 2 most common laboratory factors that suggest myocarditis. Serum troponin was abnormal in 94% of cases, and the degree of troponin elevation was also a reasonable predictor of adverse events.^[16]^ BNPs are markers of myocardial stretch and should be examined in symptomatic patients, but they may also be normal examination phenotype.^[17]^ Among patients with immune myocarditis, 66% had abnormal BNP, 89% had abnormal ECG, and 51% had normal left ventricular ejection fraction (LVEF).^[16]^ Most patients with immune myocarditis have abnormal ECGs at the initial presentation, but normal ECGs does not rule out immune myocarditis. ECG changes are extensive and include sinus tachycardia, conduction abnormalities, QRS/QT prolongation, and arrhythmias. ECG changes are dynamic (change from baseline) over a time frame consistent with the onset of immune myocarditis. Echocardiography is the standard first-line test for the evaluation of immune-related myocarditis, owing to its wide availability and ease of performance. However, even in fulminant myocarditis, LVEF may be normal, and a normal LVEF does not rule out major adverse cardiac events. Since echocardiography is not specific for immune myocarditis, it lacks sensitivity in the case of relatively preserved systolic function.^[17]^ Cardiovascular magnetic resonance (CMR) is the imaging method of choice for the diagnosis of immune myocarditis and has several distinct advantages over echocardiography. The main advantage of CMR is the tissue characterization technique, which can be used as a surrogate for myocardial injury.^[18–20]^ The sensitivity of CMR for immune myocarditis is 76% and the specificity is 96%.^[18,20]^ Endomyocardial biopsy (EMB) is the gold standard for the diagnosis of myocarditis; however, it is underutilized owing to its invasiveness and associated potential complications.^[21]^ The clinical manifestations of immune myocarditis are extensive and include chest pain, palpitations, chronic or acute heart failure, and pericardial effusion.

In general, permanent discontinuation of ICIs therapy is recommended in patients with severe IRAEs. Discontinuation of ICI therapy and immunosuppression are cornerstones of the management of ICI-related myocarditis, and the timing of treatment may be important given the potential for rapid progression to fatal cardiovascular events.^[22]^ Therefore, immediate initiation of immunosuppression is recommended, even without further confirmatory testing. In the acute phase, high-dose corticosteroids should be considered as the first-line therapy. Apart from treatment with high-dose corticosteroids, there are few data on optimal follow-up treatment if steroids fail. If high-dose steroids do not resolve myocarditis, infliximab therapy should be considered. If the patient is unstable, intravenous immunoglobulin, antithymocyte globulin, and plasma exchange should be considered.^[23]^ In stable patients, either biopsy-proven with severe myocarditis or refractory to corticosteroid therapy, other options should be considered tacrolimus or mycophenolate mofetil as immunosuppressants for cardiotoxicity.^[24]^ Standard antiheart failure and antiarrhythmic therapy should also be initiated. Both abatacept and alemtuzumab are possible treatments for ICI-induced myocarditis, whereas infliximab increases the risk of death from cardiovascular causes and should be avoided.^[25]^ The development of immune myocarditis is especially challenging due to its potential impact on cancer management and patient outcomes.

Based on the severe adverse outcomes of immune myocarditis, biomarkers for predicting toxicity are particularly important in patients with TET, who are currently considering immunotherapy. A study found that patients with thymoma who developed immune-related myocarditis after receiving avelumab had detectable titers of acetylcholine receptor (AChR)-binding autoantibodies before starting treatment, whereas patients without myocarditis had no AChR autoantibodies before treatment.^[26]^ Besides, patients who developed IRAEs also had severe B cell reduction and lower levels of conventional dendritic cells and regulatory T cells.^[26,27]^ Although careful consideration of the clinical history and the development of predictive biomarkers may reduce the risk of IRAEs in TET patients, these risks are unlikely to be eliminated owing to the underlying biology of these diseases. Therefore, it is vital to develop other risk-mitigation strategies, such as the prophylactic use of immunosuppressants in combination with immunotherapy, to rechallenge patients who have previously experienced immunotoxicity. In a small series of five patients with solid tumors, immune-related enterocolitis developed after ICIs monotherapy or combination therapy and concomitant use of infliximab, a tumor necrosis factor (TNF)-α inhibitor, and ICIs was safe and prevented further flare-ups of immune-related enterocolitis.^[28]^ Confirmation of these results in larger clinical trials and assessment of new biomarkers is expected to improve the safety of immunotherapy in patients with TETs and to identify those most likely to benefit from treatment.

## 4. Conclusions

Due to the thymic origin, the incidence of IRAEs in TETs is much higher than that in other cancer types, particularly immune myocarditis and myasthenia gravis. Furthermore, compared with thymic carcinoma, thymoma is often accompanied by ADs associated with T cell-mediated autoimmunity, especially myasthenia gravis. Immune myocarditis is a rare, but often severe, toxic effect. Our report provides a new reference for the occurrence, timing, treatment, and prognosis of patients with immune myocarditis and emphasizes the urgent need for further research. In future studies, it would be interesting to explore markers that predict IRAEs, alert physicians to avoid delaying timely treatment initiation, and ultimately determine which populations would benefit from ICIs without severe IRAEs.

## Author contributions

**Conceptualization:** Guikai Ma, Guohua Yu.

**Data curation:** Guikai Ma, Hui Wang.

**Project administration:** Guohua Yu, Jun Chen, Wenjing Song.

**Resources:** Hui Wang.

**Supervision:** Wenjing Song.

**Validation:** Jun Chen.

**Writing – original draft:** Shiwei Liu.
